# Burnout and metabolic syndrome among different departments of medical center nurses in Taiwan‐Cross‐sectional study and biomarker research

**DOI:** 10.1002/1348-9585.12188

**Published:** 2021-01-19

**Authors:** Meng‐Ting Tsou, Tsung‐Ping Pai, Te‐Ming Chiang, Wei‐Hsin Huang, Hsiu‐Mei Lin, Shu‐Chen Lee

**Affiliations:** ^1^ Department of Family Medicine Mackay Memorial Hospital Taipei Taiwan; ^2^ Department of Occupation Medicine Mackay Memorial Hospital Taipei Taiwan; ^3^ Mackay Junior College of Medicine, Nursing, and Management New Taipei Taiwan; ^4^ Department of Family Medicine and Occupation Medicine Lian‐Xin Clinic Taoyuan Taiwan; ^5^ Department of Medicine Mackay Medical College New Taipei Taiwan; ^6^ Department of Occupational Safety &Health Mackay Memorial Hospital Taipei Taiwan; ^7^ Department of Community Health Center Mackay Memorial Hospital Taipei Taiwan

**Keywords:** biomarker, burnout, metabolic syndrome, nurses, Taiwan

## Abstract

**Objectives:**

The cross‐sectional study aimed to analyze the association between burnout, work‐related factors, and metabolic syndrome (Mets) in nurses from several departments of a tertiary hospital in Taiwan. Exploring biomarkers could provide for prevention.

**Methods:**

Demographic data were obtained through a written questionnaire and include the following information: gender, age, education level, psychosocial and work situations, such as departments, working hours, work shift, depression, and sleep time. Burnout was evaluated according to the Chinese Burnout inventory, Mets was evaluated according to the criteria of the National Cholesterol Education Program of Taiwan—Treatment Panel for Adults III (NCEP‐ATP III).

**Results:**

A total of 1758 nurses participated with a median age of 35.2 years. The prevalence of burnout and Mets was 6.4% and 13.84%, respectively. The results showed that burnout induced higher risk of Mets, odds ratio (OR) 1.70 (95% confidence interval, 1.04‐3.05). Other factors, such as out‐patient nurses, seniority (4‐10 and >10 years), working hours (51‐59 h/wk), nigh shift, Brief Symptom Rating Scale‐5 (score 10‐14 and ≧15), poor self‐rated health status, and inadequate sleep time, led to higher risk of Mets. Biomarkers research showed that Glycated hemoglobin (Hba1c) was significantly associated with burnout nurses (OR = 24.72, *P* < .001), but thyroid‐stimulating hormone and free thyroxin were not.

**Conclusions:**

Results suggested positive associations between burnout and Mets in nurses. For nurses with higher seniority, long hours of work, night shifts, poor physical and mental conditions, and poor lifestyle habits in different departments, strategies are needed to prevent burnout and Mets.

## 1 INTRODUCTION

Burnout, defined as a psychological syndrome derived from prolonged exposure to chronic stressors at work, is often observed in healthcare workers.^1^ A study in Taiwan observed the prevalence of high work‐related burnout was nurses (66%), physician assistants (61.8%), and physicians (38.6%).^2^ Previous studies have shown that Mets are prevalent in hospital workers, the prevalence was up to 38.7% in Mexico female nurses,^3^ and 24.4% in Brazil primary healthcare nursing professionals.^4^ In Taiwan tertiary hospital study showed that physicians had the highest prevalence of Mets (18.3%) and nurse had the lowest prevalence (6.6%).^5^

Hospital staff, special nurses, who faced with the heavy workload, stress of caring for patients, long working hours, and shifts appeared to be at a higher risk of adverse health effects, which include Mets and burnout.^3^, ^4^, ^5^, ^6^

One study had analyzed the association between burnout and Mets in primary health care nurses, the results showed that burnout syndrome increases the risk of Mets by 1.45 (95% CI = 1.17‐1.81).^4^ Another study mentioned that associations of burnout syndrome domains, including emotional exhaustion (aOR: 14.95; 95% CI: 1.5‐148.7), personal accomplishment (aOR: 0.13; 95% CI: 0.01‐0.99), and night shift (aOR: 12.39; 95% CI: 1.02‐150.5) with increased waist circumference (WC).^3^ However, such studies have seldom compared the burnout and Mets prevalence across different nurse departments.

Nurses faced many different physical and psychological stressors, such as heavy workload, physical exhaustion caused by caring for patients, reduced relative physical activity; as well as, anxiety and depression caused by working long hours and experiencing heavy stress.^7^, ^8^, ^9^ Many studies report that nurses had chronic occupational stress, low levels of self‐care and shift work interferes, making it difficult for these professionals to develop healthy habits,^7^, ^8^, ^9^ and higher prevalence of Mets.^10^, ^11^, ^12^

Literature searches show a lack of unequivocal conclusions about the association of burnout with stress biomarkers. In a systematic review and meta‐analyses of 31 studies on 38 biomarkers, the results showed that there are no potential biomarkers in burnout due to the incompatibility of the studies designs and methods (included the characters of patients, assess biomarkers, and control for confounders).^13^

But there are still many possible biological mechanisms between burnout and Mets that are discussed. First, chronic stress is associated with hyperstimulation of the hypothalamic‐pituitary‐thyroid axis (HPT) and, as a consequence, increases cortisol secretion (included salivary cortisol),^14^, ^15^ and relationship with thyroid function (included TSH, T3, and T4).^16^ Asberg et al found that there were significant differences in T3 and TSH among participants from sick leave groups, occupational stress groups, and healthy groups.^17^ TSH was an early and sensitive biochemical predictor of chronic stress with a significant increase in stress events in Harbeck et al study.^18^ But Guo Y et al^16^ found no significant association between burnout symptoms and TSH, T3, and T4 that are consistent with existing literature on both burnout and biochemical markers.^13^, ^19^ Second, sympathetic‐adrenal‐medullary axis stimulates adrenal glands to release catecholamine (eg, epinephrine and norepinephrine).^20^, ^21^

Third, the study of Grossi et al proved that high burnout in women is associated with high levels of Hba1c.^22^ The results from physicians in Bulgaria showed that age, saliva cortisol, and blood concentration of Hba1c are significantly associated with burnout syndrome.^15^ Elevated Hba1c can be regarded as a marker for the Mets.^22^ Hba1c levels increase in response to psychosocial stress, eg, job strain, prolonged working time, and decrease in response to ameliorations in psychosocial conditions.^23^

In the previous studies, it was proved that burnout increases the risk of Mets in primary healthcare nurses,^4^ but as we know, there was not researches mentioned this hypothesis in medical centers, only one study proposed that labor domains increased the risk of Mets domains.^3^ Therefore, our hypothesis in this study mainly included to prove the relationship of burnout and other work factors with Mets in tertiary medical center. On the other hand, after evaluating the influence of work‐related factors, we would like to discuss whether lifestyle factors were playing another main role that increased the risk of Mets among different departments.

The purposes of this research were: (a) to analyze the association between burnout and Mets in nurses from several departments of a medical center in northern Taiwan; (b) to analyze the association between burnout and other work‐related factors with the individual factors of Mets; and (c) exploring biochemical markers of burnout and Mets which could help to understand physiological changes and may provide useful evidence for preventing burnout symptoms.

## 2 MATERIALS AND METHODS

### 2.1 Study design and participants

The observational, cross‐sectional study was conducted in the Northern Region of Taiwan, at Mackay Memorial Hospital, a 2000‐bed tertiary hospital. This region has an estimated population of 2.67 million. Metabolic syndrome (Mets) data were obtained from routine examinations by nursing professionals.

The questionnaires were collected between December 2018 and March 2019. Nursing departments included outpatient clinics, emergency rooms, general wards, intensive care units, operation rooms, and dialysis clinics. A total of 1767 nurses were invited to participate, and nine cases were excluded due to incomplete questionnaires. The actual response rate was 99.5%.

#### 2.1.1 Socio‐demographic data and working information

The information was obtained through structured questionnaires, which were designed according to the Institute of Labor, Occupational Safety, Ministry of Labor (Survey of Perceptions of Safety and Health in the Work Environment in 2016 Taiwan; ILOSH105‐M309),^24^ including five major fields: (a) basic data and socioeconomic status (included age, education level, department, seniority, working hours, work style); (b) personal lifestyle (included sleep duration, smoking, alcohol, exercise, fruit and vegetable intake); (c) physical work environment (included exposure to dust, temperature, radiation, needle stinge, and so on); (d) psychosocial work environment (included Chinese occupational burnout inventory and Maslach Burnout Inventory–Health Services Survey test); (e) physical and mental health status (included Nordic Musculoskeletal Questionnaire and Brief Symptom Rating Scale [BSRS]). We issued 180 questionnaires in advance and 168 questionnaires were recovered and the recovery rate was 93.33%. Analysis of the internal consistency reliability of each part of the scale, showed the Cronbach α to be between 0.82 and 0.93, having good reliability.

#### 2.1.2 Anthropometric measurements

We collected baseline characteristics and anthropometric measurements, including age, body height, body weight, WC, and blood pressure (BP). Height was measured with a standard stadiometer with an approximation value of 0.01 cm. Weight was measured using a set of standard calibrated electronic scales, volunteers were in light clothing with an approximate value of 0.01 kg. To measure WC, the anatomical region was used: the midpoint between the lowest rib and the upper point of the iliac crest and at the end of normal expiration. Standardized sphygmomanometer cuff‐defined BP values were measured while resting.

Anthropometric measurements (height, weight, WC and BP) were collected and recorded by trained nurses who did not have access to patient information in a laboratory center.

#### 2.1.3 Laboratory data acquisition and analysis

Free T4 (fT4), and TSH (DiaSorin, Saluggia, Italy) were determined by an immunoradiometric assay using a commercial kit. All the participants had no thyroid history (from history taking) and no acute or serious health problems, potential confounding by nonthyroidal illness. The serum for measurement was drawn after 12 hours of fasting.

A Hitachi 7170 Automatic Analyzer (Hitachi Corp., Hitachinaka Ibaraki, Japan) was used to measure the levels of fasting glucose, Hba1c (hexokinase method), total cholesterol, and triglyceride. The analyzes of lipid markers, including low and high density lipoprotein cholesterol (homogeneous enzymatic colorimetric assay), were analyzed using the Hitachi 7170 automatic equipment (Hitachi Corp.)

#### 2.1.4 Burnout assessment

Burnout domains evaluation were assessed using two systems: one was the Chinese occupational burnout inventory,^25^ and the other was the Maslach Burnout Inventory–Health Services Survey test^26^ (Cronbach's α: 0.84 in Chinese version).^27^ The questionnaire of the Chinese occupational burnout inventory consisted of three domains: psychological work demands, job control (personal accomplishment), and employment stability. Each domain had its total points divided into tertiles.^25^ The Cronbach's α was shown as 0.84‐0.91 in every domain, which means that this questionnaire has good reliability and validity.^25^

The Maslach Burnout Inventory evaluated three domains: emotional exhaustion, depersonalization, and personal accomplishment (equal to job control in Chinese occupational burnout inventory), and if emotional exhaustion score ≧27, depersonalization score ≧13, and personal accomplishment ≦31 was categorized as high job burnout.^26^ The psychological work demands, depersonalization, and emotional exhaustion showed direct relation to burnout, while, on the contrary, job control (personal accomplishment) and employment stability showed a negative association.^25^, ^26^

In this study, we used the Chinese occupational burnout inventory for classification into the burnout and non‐burnout groups. Burnout was defined as the third tertile of psychological work demands plus the first tertile of job control and the first tertile of employment stability, according to the design of professor Cheng.^25^

#### 2.1.5 Classification according to the Mets and thyroid function

To analyze the metabolic score, we used the classification of the National Cholesterol Education Panel of the National Treatment Program for Adults III (NCEP‐ATP III) with specific cut‐off points for the Taiwanese population for abdominal obesity.

Individuals who met the Mets criteria with at least three of the following five components were defined as having the Mets: (a) WC ≥90 cm for men and 85 cm for women; (b) high density lipoprotein cholesterol (HDL‐C) <40 mg/dL for men and <50 mg/dL for women; (c) triglyceride (TG) levels ≥150 mg/dL; (d) BP ≥130/85 mmHg or treatment for hypertension (HTN); and (e) fasting blood glucose (SFB) ≥100 mg/dL or treatment for type 2 diabetes mellitus (DM).^28^ The reference ranges for fT4, and TSH were 1.0‐1.71 ng/dL, and 0.4‐4.0 mIU/mL respectively.^29^ The normal value of Hba1c is about 4%‐6%.^22^

#### 2.1.6 Brief Symptom Rating Scale‐5

Mental health was analyzed using the BSRS‐5. It is a self‐assessment questionnaire, which includes five items, and requires respondents to report whether they felt tense, blue, irritated, or inferior or if they had any problems falling asleep in the past week. Responses are rated on a scale of 0 to 4, with 0 being "nothing" and 4 being "extremely". The total score ranges from 0 to 20.^30^, ^31^

The internal consistency was analyzed by Cronbach's α coefficient, the BSRS‐5 coefficients were from 0.77 to 0.90.^30^ The test‐retest reliability coefficient showed a result of 0.82. When using a score ≥6 as the cut‐off point for psychiatric cases, the BSRS‐5 accurate classification rate was 76.3% (sensitivity of 78.9%, specificity of 74.3%, positive predictive value of 69.9% and negative predictive value of 82.3%)^30^ and was divided into four groups: “no symptoms” (0‐5), “mild” (6‐9), “moderate” (10‐14), and “severe ”(Over 15).^30^, ^31^

### 2.2 Ethics approval

The study protocol was evaluated and approved by the Human Research Ethics Committee of Mackay Memorial Hospital (project research number 18MMHISO150).

All participants provided written informed consent. Confidentiality in data collection was preserved, taking into account ethical issues, such as autonomy and respect for people. All the guidelines of the Declaration of Helsinki have been performed. To ensure data confidentiality, patient identification information was replaced by a folio number.

### 2.3 Statistical analysis

We performed a descriptive analysis to characterize the population sample. These data are presented as mean ± standard deviation for continuous variables; numbers and percentages are presented for categorical variables.

According to the cut‐off point of the NCEP‐ATP III, the Mets factors were classified into two categories. For each factor, frequencies and percentages were calculated.

For comparisons between the groups, Student's *t*‐test was used to analyze continuous variables; the chi‐square test was used for categorical variables. Univariate logistic regression analyses were performed to investigate the possible associations between Mets and burnout, work‐related factors, emotional symptoms, or life‐style factors. Multivariate logistic regression analyses were then performed to adjust for age and other variables that showed significant association in the univariate analyses. For multivariate logistic regression analysis, the Mets factors were analyzed separately as dependent variables, and the burnout, work‐related factors, and emotional symptoms were evaluated as independent variables. Multivariate logistic regression model was used for biomarker analysis.

We performed all analyses using SPSS 22 (IBM Corp., Armonk, NY, USA) for Windows. We considered two‐sided *P* < .05 to be statistically significant.

## 3 RESULTS

### 3.1 1 Characteristics of the participants

A total of 1758 subjects, including 113 burnout nurses (6.4%) and 1645 non‐burnout nurses (93.6%) according to Chinese occupational burnout inventory, were assessed [125 burnout (7.1%) and 1633 non‐burnout (92.9%) according to Maslach Burnout Inventory]. There was no statistical difference between these groups in age, gender, education level, seniority at work, working style, sleep duration, and personal lifestyle (exercise, smoking, alcohol, and fruit and vegetable intake). A higher proportion of burnout nurses worked in outpatient clinics, emergency rooms, operation rooms, and dialysis room. Longer working hours per week (>45 h/wk), higher BSRS‐5 scores (>6), poor self‐rated health status, and inadequate sleep by self‐assessment increased the proportion of burnout (*P* < .01). Scores for different domains of the Chinese occupational burnout inventory and the Maslach Burnout Inventory in the burnout group were higher than those in the non‐burnout group (*P* < .001).

There is no statistical difference in the average of the five Mets factors in the burnout and no‐burnout groups. The prevalence of Mets was higher in the burnout group (25.58% vs 12.91%, *P* = .001). A higher abnormal proportion of Mets factors was found in the burnout group (except central obesity). The biomarkers, including Hba1c, fT4, and TSH were no statistical difference. The characteristics of participants were shown in Table 1.

**TABLE 1:** Demographic and clinical characteristics of study participants (n = 1758) across burnout syndrome

	Total (n = 1758)		Burnout (n = 113)		No‐ burnout (n = 1645)		*P* value
Age, y (mean, SD)	35.20 (11.02)		36.68 (11.06)		35.10 (11.02)		.12
Female gender, n (%)	1685	95.85	109	96.46	1576	95.81	.74
Education level, n (%)							
≦Senior high school	45	2.56	5	4.42	40	2.43	.07
College	1608	91.47	106	93.81	1502	91.31	
≧Graduate School	105	5.97	2	1.77	103	6.26	
Department, n (%)							<.001
Out‐patient	375	21.33	31	27.43	344	20.91	
Emergency	129	7.34	16	14.16	113	6.87	
General ward	865	49.20	46	40.71	819	49.79	
Intensive care unit	241	13.71	5	4.42	236	14.35	
Operation room	113	6.43	12	10.62	101	6.14	
Dialysis	35	1.99	3	2.65	32	1.95	
Seniority, n (%)							.24
<2 y	345	19.62	14	12.39	331	20.12	
2‐4 y	276	15.70	21	18.58	255	15.50	
4‐10 y	532	30.26	37	32.74	495	30.09	
>10 y	605	34.41	41	36.28	564	34.29	
Working hours/wk, n (%)							.002
≦45 h	1041	59.22	54	47.79	987	60.00	
46‐50 h	580	32.99	41	36.28	539	32.77	
51‐59 h	101	5.75	15	13.27	86	5.23	
≧60 h	36	2.05	3	2.65	33	2.01	
Work style, n (%)							.43
Regular class	643	36.58	38	33.63	605	36.78	
Night shift	116	6.60	5	4.42	111	6.75	
Three shifts	999	56.83	70	61.95	929	56.47	
BSRS‐5 (mean ±SD)	5.71 ± 3.92		8.39 ± 4.79		5.52 ± 3.79		<.001
BSRS‐5, n (%)							<.001
≦5	1021	58.08	38	33.63	983	59.76	
6‐9	435	24.74	30	26.55	405	24.62	
10‐14	242	13.77	28	24.78	214	13.01	
≧15	60	3.41	17	15.04	43	2.61	
Self‐rated health status, n (%)							<.001
Good	443	25.20	13	11.50	430	26.14	
Common	1112	63.25	70	61.95	1042	63.34	
Bad	203	11.55	30	26.55	173	10.52	
Sleep duration, h (mean, SD)	6.61 (1.10)		6.34 (1.15)		6.63 (1.10)		.19
Self‐assessment of sleep time							.02
Adequate	655	37.30	30	26.55	625	37.99	
Inadequate	1101	62.70	83	73.45	1018	61.88	
Exercise, n (%)	828	47.10	49	43.36	779	47.36	.41
Smoking, n (%)	49	2.79	2	1.77	47	2.86	.50
Drink, n (%)	103	5.86	7	6.19	96	5.84	.86
Chinese occupational burnout inventory (mean, SD)							
Psychological work demands	74.53 (16.69)		95.04 (5.48)		73.12 (16.27)		<.001
Job control	57.06 (10.98)		41.79 (8.76)		58.11 (10.31)		<.001
Employment stability	60.86 (14.91)		37.46 (14.27)		62.47 (13.54)		<.001
Maslach Burnout Inventory (mean, SD)							
Emotional exhaustion	18.79 (7.38)		24.39 (5.18)		17.40 (7.25)		<.001
Depersonalization	4.60 (2.38)		5.92 (2.56)		4.51 (2.31)		<.001
Personal accomplishment	33.72 (6.49)		24.69 (5.18)		34.34 (6.09)		<.001
Metabolic syndrome, n (%)	163	13.84	22	25.58	141	12.91	.001
WC, cm (mean ± SD)	75.22 (10.43)		76.12 (11.09)		75.16 (10.38)		.35
SBP, mmHg (mean, SD)	116.44 (14.43)		117.00 (13.85)		116.39 (14.48)		.70
DBP, mmHg (mean, SD)	67.33 (10.24)		67.76 (10.08)		67.03 (10.25)		.68
BS, mg/dL (mean, SD)	93.62 (15.88)		94.11 (13.90)		93.58 (16.01)		.74
TG, mg/dL (mean, SD)	86.40 (14.99)		88.50 (12.22)		86.25 (15.79)		.72
HDL‐C, mg/dL (mean, SD)	61.92 (14.33)		62.51 (14.26)		61.88 (14.34)		.68
Central obesity^a^	486	27.98	37	32.74	449	27.65	.24
Elevated BP^b^	273	20.00	31	31.96	242	19.09	.002
Hyperglycemia^c^	319	18.34	32	28.57	287	17.64	.004
Hypertriglyceridemia^d^	200	11.49	22	19.64	178	10.93	.005
Low HDL‐C^e^	271	17.75	27	26.73	244	17.11	.014
Biomarkers							
Hba1c, % (mean, SD)	5.47 (0.59)		5.44 (0.52)		5.47 (0.60)		.87
Free T4, ng/dL (mean, SD)	1.20 (0.33)		1.16 (0.24)		1.23 (0.40)		.53
TSH, mU/L (mean, SD)	2.04 (1.63)		2.15 (1.64)		2.03 (1.63)		.75

Note

Smoking status (current or past/never), alcohol consumption (0‐1 drinks per wk/≧2 drinks per wk), exercise (≧3 times/wk, ≧30 mins/time).

Abbreviations: BP, blood pressure; BS, blood sugar; BSRS, Brief Symptom Rating Scale; free T4, free thyroxine; Hba1c, hemoglobin a1c; HDL‐C, high density lipoprotein‐cholesterol; TG, triglyceride; TSH, thyroid‐stimulating hormone; WC, waist circumference.

^a^ WC ≥90 cm in men or ≥80 cm in women.

^b^ SBP ≧ 130 mmHg or DBP ≧ 85 mmHg or self‐reported hypertension.

^c^ Fasting blood glucose ≥100 mg/dL or self‐reported diabetes mellitus.

^d^ TG: TG ≥ 150 mg/dL.

^e^ HDL‐C <40 mg/dL in men or <50 mg/dL in women.

### 3.2 The prevalence of Mets and Mets factors in different departments nurses with burnout syndrome

For the population sample studied, the prevalence was 25.58% for nurses with burnout and Mets. According to the Mets criteria, 32.74% of nurses with burnout had increased WC, followed by high BP (31.96%), high fasting plasma glucose (28.57%), and low HDL‐C (26.73%). Only 19.64% had high triglyceride (Table 1).

Table 2 showed the prevalence of Mets and Mets factors in different department nurses with burnout syndrome (excluded emergency and dialysis nurses for Mets participants were less, five persons). The results presented that the out‐patient department had the highest prevalence of Mets and Mets factors compared with other departments.

**TABLE 2:** The prevalence of Mets and Mets factors in different departments nurses with burnout syndrome

	Out‐patient	General ward	Intensive care unit	Operation room	Mantel‐Haenszel *P* value
	Burnout				
	n (%)	n (%)	n (%)	n (%)	
Metabolic syndrome	11 (11/61, 18.03%)	7 (7/74, 9.46%)	1 (1/13, 7.69%)	1 (1/8, 12.5%)	.04
Metabolic components					
Elevated blood pressure^a^	13 (15.12%)	11 (9.82%)	2 (6.06%)	2 (10.53%)	.01
Central obesity^b^	11 (8.33%)	18 (7.41%)	1 (1.89%)	1 (5.56%)	.03
Hyperglycemia^c^	18 (13.74%)	7 (6.19%)	2 (6.25%)	2 (11.11%)	.03
Hypertriglyceridemia^d^	11 (14.10%)	7 (8.54%)	1 (7.14%)	1 (8.33%)	.03
Low HDL‐C^e^	8 (13.11%)	12 (8.11%)	1 (4.00%)	1 (8.33%)	.02

Note

Excluded the data of emergency and dialysis nurses (the Mets participant was < 5 persons).

^a^ SBP ≧ 130 mmHg or DBP ≧ 85 mmHg or self‐reported hypertension.

^b^ Waist circumference ≥90 cm in men or ≥80 cm in women.

^c^ Fasting blood glucose ≥100 mg/dL or self‐reported diabetes mellitus.

^d^ TG ≥ 150 mg/dL.

^e^ HDL‐C <40 mg/dL in men or <50 mg/dL in women.

### 3.3 The risk of burnout and working factors with Mets

Logistic regression analysis was performed to estimate the crude and multivariable‐adjusted odds ratios and to identify burnout and working factors associated with Mets (Table 3). Multivariable model included burnout, age, and independence of risk factors in the univariate analysis. Age, gender, and factors that were significantly associated with emotional symptoms were included in the multiple logistic regression model. The results showed that burnout led to higher risk of Mets, 1.70 (95% CI = 1.04‐3.05). Other factors, such as out‐patient department; seniority at work (4‐10 years and >10 years); working hours (especial 51‐59 h/wk); night shift; BSRS‐5 (score 10‐14 and ≧15); poor self‐rated health status; and inadequate self‐assessment of sleep time, incurred statistical significance in the higher risk of Mets (*P* < .05).

**TABLE 3:** Univariate and multivariate logistic regression analysis of burnout and working factors influencing Mets

Variables	Crude			Mulivariable		
	OR	95% CI	*P* value	OR	95% CI	*P* value
Burnout	2.32	1.38‐3.88	.001	1.70	1.04‐3.05	.04
Age	1.06	1.04‐1.07	<.001	1.05	1.02‐1.08	.001
Education level						
≦Senior high school	1	—	—	1	—	—
College	0.27	0.12‐0.57	.001	0.74	0.32‐1.70	.48
≧Graduate School	0.58	0.22‐1.51	.26	0.74	0.27‐2.07	.57
Departments						
Operation room (ref)	1			1		
Out‐patient	3.33	1.04‐2.65	.002	2.78	1.05‐1.88	.02
General ward	1.37	1.08‐4.60	<.001	1.84	0.55‐1.75	.18
Intensive care unit	0.86	1.01‐5.48	<.001	1.33	0.25‐1.46	.09
Seniority						
<2 y	1	—	—	1	—	—
2‐4 y	0.69	0.35‐1.38	.29	0.60	0.30‐1.23	.16
4‐10 y	2.32	1.40‐3.85	.001	1.22	1.06‐2.21	.04
>10 y	4.06	2.52‐6.54	<.001	1.42	1.02‐2.59	.03
Working hours/wk						
≦45 h	1	—	—	—	—	—
46‐50 h	1.01	0.70‐1.46	.95	0.94	0.64‐1.56	.84
51‐59 h	1.55	0.83‐2.89	.17	1.35	1.02‐2.11	.04
≧60 h	0.80	0.24‐2.72	.72	0.72	0.13‐2.56	.56
Work style						
Regular class	1	—	—	1	—	—
Night shift	1.18	0.65‐2.14	.59	2.99	1.45‐6.15	.003
Three shift	0.45	0.32‐0.64	<.001	1.21	0.70‐2.10	.50
BSRS‐5						
≦5	1	—	—	—	—	—
6‐9	1.21	0.81‐1.83	.36	1.33	0.86‐2.05	.52
10‐14	1.85	1.19‐2.87	.01	2.50	1.54‐4.16	.02
≧15	2.08	1.00‐4.36	.05	2.66	1.10‐4.78	.03
Self‐rated health status						
Good	1	—	—	1	—	—
Common	1.65	1.04‐2.61	.03	1.43	0.88‐2.33	.15
Bad	3.62	2.06‐6.35	<.001	3.28	1.76‐6.13	<.001
Self‐assessment of sleep time						
Adequate	1	—	—	—	—	—
Inadequate	1.46	1.14‐2.08	.02	1.24	1.01‐1.78	.04
Exercise (no vs yes)	0.99	0.71‐1.38	.95	—	—	—
Smoking (yes vs no)	0.86	0.30‐2.47	.77	—	—	—
Drink (yes vs no)	0.71	0.33‐1.50	.37	—	—	—

Note

Multivariable model included: burnout, age and independence of risk factors in the univariate analysis.

In Table S1, multivariable logistic regression analysis was performed to identify burnout, age, education level, and working factors associated with Mets in different department (excluded emergency and dialysis nurses for Mets participants were less, five persons). The result showed that burnout increased Mets risk in out‐patient department (OR: 2.67, 95% CI: 1.03‐6.95); age induced higher Mets risk in out‐patient, general ward, and operation room departments; night shift increased higher Mets risk in out‐patient and general ward departments (*P* < .05).

### 3.4 Characteristics of the participants in different departments

In Table 4, we analyzed the age and lifestyle factors of different departments. The result showed that the age of out‐patient (45.47 ± 9.54 years) and dialysis departments (44.22 ± 8.08 years) were statistically significantly older than other departments. There was no significant difference in smoking and drinking habits in different departments However, regular exercise, and fruit and vegetable intake habits were significantly lower in out‐patient nurses comparing to other departments.

**TABLE 4:** The age and prevalence of lifestyle actors in different departments

Factors	Out‐patient (375)	Emergency (129)	General ward (865)	Intensive care unit (241)	Operation room (113)	Dialysis (35)	Total (1758)	*P* value
Age, mean (SD)	45.27 (9.54)	31.74 (8.76)	31.49 (9.17)	31.88 (9.20)	39.15 (11.64)	44.22 (8.08)	35.20 (11.02)	<.001
Exercise, n (%)	171 (45.6)	65 (50.4)	397 (45.8)	116 (48.1)	58 (51.3)	21 (61.9)	828 (47.1)	.04
Smoking, n (%)	16 (4.3)	7 (5.4)	9 (1.0)	8 (3.3)	8 (7.1)	1 (2.9)	49 (2.8)	.68
Drink, n (%)	15 (4)	18 (14)	57 (6.6)	7 (2.9)	2 (1.8)	4 (11.4)	103 (5.9)	.56
Fruit intake, n (%)	206 (54.9)	98 (76)	698 (80.7)	197 (81.7)	92 (81.4)	34 (97.1)	1456 (82.8)	.03
Vegetable intake, n (%)	351 (93.6)	122 (94.6)	825 (95.4)	237 (98.3)	108 (95.6)	35 (100)	1678 (95.4)	.01

Note

The column is the number and proportion of cases with this behavior. Fruit intake: One serving: equivalent to one medium orange, apple or guava. Vegetable intake: One serving: equivalent to 15 cm plate or more than half a bowl. Smoking status (current or past/never), alcohol consumption (0‐1 drinks per wk/≧2 drinks per wk), exercise (≧3 times/wk, ≧30 mins/time).

Table S2 showed the working factors, burnout domains, BSRS‐5 score, and sleep condition in different departments, there were statistically significance (*P* < .05).

### 3.5 Analysis of the association between burnout and working factors with the components of the Mets factors

Multivariate logistic regression analysis was performed in Table 5 that showed the nurses in burnout group had higher risks of high BP, high BS, high TG, and low HDL‐C (aOR = 1.82, 1.76, 1.80, 1.66, *P* ≤ .05). The results showed that nurses who worked for more than 10 years and had poor self‐rated health status had higher risk of having five Mets factors. Nurses with BSRS‐5 score more than 15 had a higher risk of having high TG. On the contrary, nurses working three shifts had a lower risk of having five Mets factors. Different nursing departments had an impact on the risk of different Mets factors.

**TABLE 5:** Adjusted odds ratios and 95% CI of the burnout domains and working factors with Mets factors^a^

	High WC	High BP	High BS	High TG	Low HDL‐C
Burnout					
No (ref)	1	1	1	1	1
Yes	1.15 (0.76‐1.74)	1.82 (1.13‐2.93)**	1.76 (1.08‐2.87)**	1.80 (1.07‐3.02)**	1.66 (1.04‐2.64)**
Education level					
≦Senior high school (ref)	1	1	1	1	1
College	0.93 (0.47‐1.83)	0.94 (0.42‐2.08)	0.70 (0.34‐1.46)	0.84 (0.39‐1.78)	1.10 (0.48‐1.50)
≧Graduate School	1.10 (0.51‐2.37)	0.84 (0.33‐2.16)	0.68 (0.30‐1.53)	0.70 (0.29‐1.67)	1.03 (0.41‐2.61)
Departments					
Out‐patient (ref)	1	1	1	1	1
Emergency	1.08 (0.67‐1.75)	1.01 (0.54‐1.90)	0.86 (0.46‐1.61)	0.84 (0.41‐1.70)	1.58 (0.87‐2.87)
General ward	1.14 (0.84‐1.56)	1.01 (0.67‐1.53)	0.97 (0.68‐1.39)	0.93 (0.62‐1.39)	1.71 (1.16‐2.52)**
Intensive care unit	0.78 (0.52‐1.17)	0.90 (0.54‐1.51)	0.93 (0.57‐1.52)	0.50 (0.27‐0.95)**	0.95 (0.55‐1.62)
Operation room	0.37 (0.21‐0.66)***	0.79 (0.43‐1.45)	0.46 (0.25‐0.85)**	0.59 (0.30‐1.17)	0.68 (0.35‐1.34)
Dialysis	0.40 (0.16‐1.01)	0.48 (0.17‐1.35)	0.64 (0.28‐1.49)	0.36 (0.11‐1.24)	0.88 (0.32‐2.38)
Seniority^b^					
<2 y (ref)	1	1	1	1	1
2‐4 y	0.98 (0.64‐1.48)	0.81 (0.51‐1.31)	1.03 (0.48‐2.18)	0.71 (0.29‐1.71)	0.87 (0.53‐1.42)
4‐10 y	1.86 (1.33‐2.60)***	1.07 (0.72‐1.61)	3.13 (1.78‐5.51)***	3.29 (1.81‐5.98)***	1.36 (0.90‐2.04)
>10 y	2.39 (1.72‐3.33)***	3.83 (2.58‐5.69)***	11.71 (6.82‐20.10)***	5.18 (2.89‐9.29)***	1.73 (1.18‐2.53)**
Working hours/wk					
≦45 h (ref)	1	1	1	1	1
46‐50 h	0.97 (0.77‐1.24)	0.86 (0.62‐1.18)	1.07 (0.78‐1.45)	0.95 (0.67‐1.35)	1.01 (0.75‐1.36)
51‐59 h	1.20 (0.75‐1.91)	1.42 (0.82‐2.48)	0.94 (0.47‐1.87)	1.43 (0.73‐2.78)	1.34 (0.76‐2.37)
≧60 h	1.18 (0.56‐2.49)	0.84 (0.30‐2.34)	1.63 (0.62‐4.26)	0.83 (0.24‐2.91)	0.90 (0.34‐2.41)
Work style					
Regular class (ref)	1	1	1	1	1
Night shift	1.19 (0.77‐1.85)	1.34 (0.79‐2.24)	0.62 (0.37‐1.04)	0.49 (0.24‐1.00)	1.46 (0.86‐2.49)
Three shifts	0.65 (0.52‐0.81)***	0.58 (0.43‐0.77)***	0.27 (0.20‐0.35)***	0.45 (0.33‐0.61)***	0.94 (0.71‐1.25)
BSRS‐5					
≦5 (ref)	1	1	1	1	1
6‐9	1.15 (0.89‐1.49)	1.05 (0.74‐1.48)	1.05 (0.75‐1.47)	1.43 (0.99‐2.06)	1.17 (0.85‐1.61)
10‐14	1.23 (0.89‐1.70)	1.27 (0.83‐1.93)	1.44 (0.94‐2.20)	1.53 (0.97‐2.48)	1.23 (0.83‐1.83)
≧15	1.59 (0.90‐2.82)	1.80 (0.90‐3.61)	1.86 (0.86‐4.03)	2.34 (1.06‐5.16)**	1.56 (0.78‐3.12)
Self‐rated health status					
Good (ref)	1	1	1	1	1
Common	1.22 (0.94‐1.60)	1.25 (0.89‐1.78)	1.53 (1.07‐2.17)**	1.03 (0.70‐1.52)	1.17 (0.83‐1.63)
Bad	1.59 (1.08‐2.33)**	1.96 (1.20‐3.20)**	2.24 (1.35‐3.70)**	1.67 (1.02‐2.85)**	2.01 (1.27‐3.19)**

^a^ Adjusted for sex, sleep time, alcohol, exercise, fruit and vegetable intake.

^b^ Replace age with seniority.

* *P* < .05.

** *P* < .01.

*** *P* < .001.

### 3.6 Analysis of the association between age, burnout, and biomarkers with nurse with Mets

In Table 6, multivariate logistic regression analysis was performed to identify predictive factors (independent variables, included burnout, age, an biomarkers) of nurses with Mets (dependent variable). The results showed that burnout, age, and Hba1c had a higher risk of having Mets in nurses group (burnout: OR = 1.27, 95% CI: 1.01‐1.39; age: OR = 1.04, 95% CI: 1.00‐1.09; Hba1c: OR = 24.72, 95% CI: 5.25‐116.49). The other biomarkers associated HPT axis (TSH and fT4) were no statistically significant difference with Mets in nurse group.

**TABLE 6:** Odds ratios and 95% CI of the burnout, age and biomarkers with Mets factors

Factors	OR	95% CI	*P* value
Burnout	1.27	1.01‐1.39	.04
Age	1.04	1.00‐1.09	.04
Hba1c	24.72	5.25‐116.49	<.001
TSH	1.19	0.49‐1.54	.45
Free T4	0.93	0.71‐1.21	.57

Note

Abbreviations: free T4, free thyroxine; Hba1c, hemoglobin a1c; TSH, thyroid‐stimulating hormone.

## 4 DISCUSSION

The prevalence of burnout syndrome varied among different professionals: 4.8% to 39.3% in health professionals, 54.9% to 56% in police officers, and 5.7% to 15.4% in professors.^32^ In this population sample of nurses from medical centers in Taiwan, the prevalence of burnout of 6.4% was lower than that described in other studies.^3^, ^6^ Several explanations are possible to analyze this magnitude of prevalence.

The first one was that we used the Chinese occupational burnout inventory,^25^ (Cronbach's α :0.84‐0.91, which means the questionnaire has good reliability and validity for Taiwanese),^25^ that was different from other studies that used the Maslach Burnout Inventory.^26^ The second explanation was that, in this study, participants were younger (median 35.2 years) than those in other studies.^3^, ^6^ Finally, the population sample of the study has a high level of education (higher than college) of 97.44%, and previous studies describe that women with high burnout scores had a higher frequency of low levels of education.^33^

A previous study described a population prevalence of Mets in Taiwan of 25.5% for men and 31.5% for women.^34^ In this study, the prevalence of Mets in the nursing staff of the investigated hospital (13.84%) was lower than that of the general population, but it was similar to another study conducted in a hospital in Taiwan (12.0%).^5^ This relationship between prevalence can be explained because the workers in the hospital were younger and exhibited a healthy effect on the worker. We also considered the presence of a higher education level as a variable that explains this association, since a relationship had been described that, the lower the level of education, the greater the frequency of the Mets.^3^, ^35^

A study by Mets in a general workforce population in Taiwan showed that the prevalence was 12.1%.^36^ We were able to observe that when comparing with other occupations in Taiwan, the prevalence of Mets among hospital staff was not low. This meant that although hospital nurses had more knowledge of Mets than the general population, the risks remained high. This finding may be associated with work stress of hospital nurses in Taiwan.^36^

In analyzing a study on stress at work and burnout of health workers at another hospital in Taiwan, we found that more than half of the employees worked shifts and worked long hours. The burnout rate of nurses was >50%.^2^ Several studies have described that work stress and long working hours, which could lead to reducing sleep and exercise, as well as changes in eating habits, can be highly associated with the Mets.^5^, ^37^, ^38^, ^39^ Studies in populations in Japan and Taiwan describe that long hours of work (>10 h/d) increased the risk of Mets and cardiovascular diseases.^38^, ^39^ Study in Mexico tertiary hospital nurses showed that working in the night shift, and labor seniority ≥15 years were associated with adiposity‐based chronic disease.^40^ In our study, we found that nurses with long working hours (51‐59 h/wk), night shift, and self‐assessment of inadequate sleep time had a higher risk of Mets, this result was consistent with previous studies.^38^, ^39^, ^40^

Other factors that can contribute to the Mets in nurses were depressed mood and stress caused by work stress.^41^ We analyzed that Taiwanese nurses had greater pressure at work and higher rates of depression (15.0%) than the general population (3.7%).^41^ In our study, we found that nurses with BSRS‐5 score 10‐14 and ≧15 and poor self‐rated health status had a higher risk of Mets, which was compatible with the mental and physical risk factors of Mets.^40^, ^41^

Previous study observed that the prevalence and risk of Mets has an increasing association with increasing age.^28^ In our study, we used seniority years instead of age and we found the same results; nurses with higher seniority (4‐10 years and >10 years) had higher risk of Mets. We analyzed the age of different departments, the result showed that the age of out‐patient (45.47 ± 9.54 years) and dialysis departments (44.22 ± 8.08 years) were statistically significantly older than other departments, which played the role to lead the out‐patient department nurse with higher risk of Mets.

Study in Mexico tertiary hospital nurses showed that unhealthy lifestyle (included less of 3 days per week and/or less of 30 minutes per session of physical activity and poor dietary habits), which were associated with adiposity‐based chronic disease.^40^ Our study showed that regular exercise, fruit and vegetable intake habits were significantly lower in out‐patient nurses compared to other departments, which were the other reasons for out‐patient department with statistically significance in higher risk of Mets. As separate studies have revealed that sedentary lifestyles may be a risk factor for Mets,^42^, ^43^ the fact that the administrative staff in another Taiwan tertiary hospital had a higher prevalence of Mets than medical technicians may be attributable to their sedentary style.^5^

The components that showed the highest prevalence of Mets with burnout in our study were central obesity (32.7%) and high BP (31.90%). We observed results similar to a study in Taiwan and other international studies.^3^, ^5^, ^35^ Therefore, adequate control of WC and BP is necessary to improve the health status of hospital workers.

In addition, values above the cut‐off: WC, BP, blood sugar, and lipid profile were lower in those who worked three shifts (*P* < .001) and higher risk of WC, BP, and lower HDL‐C in those who worked the night shift (*P*‐value borderline statistical significance). Other studies have reported similar findings, as working the night shift is associated with a greater risk of increased WC, obesity, cardiovascular disease, and cancer.^3^

To our knowledge, the present study about burnout, Mets, and their relationship with stress biomarkers (Hba1c and thyroid function) among nurses is first research. The results showed that burnout and Hba1c had a higher risk of having Mets in nurse group. Elevated Hba1c can be regarded as a marker for the Mets and burnout.^15^, ^22^ Stress‐related disturbances in HPT axis activity have an important role in the development of Mets and burnout.^15^, ^44^ Hba1c involved in the genesis of oxidative stress, which was an imbalance between prooxidants and antioxidants, in favor of the former. Previous studies indicate that oxidative stress is a concomitant of burnout in women.^22^, ^44^ Thus, our data confirmed the significant predictiveness of Hba1c in burnout and Mets, which was consistent with the association between burnout and Hba1c by other studies.^22^, ^23^

There was no significant association between burnout and thyroid function (TSH and fT4) in our study, which were consistent with existing literature on both burnout and biochemical markers.^13^, ^19^ In accordance with previous study,^45^ thyroid hormone abnormalities usually occurred after stress‐related events through the HPT axis regulation. Several studies reported that HPT axis responsive to serious stressful events, but cannot be activated by minor stress.^46^

One of the strengths of this study was that we analyzed the association between burnout and Mets in the nursing staff among different departments and the scientific literature, few studies were observed. We used the Chinese occupational burnout inventory to assess burnout, which was modified from the Maslach Burnout Inventory has good reliability and validity for workers in Taiwan. Moreover, we had a high actual response rate of 99.5%, and all participants were nurses, while a 33% participation was reported in other studies.^3^

Our research found that traditional risk factors related to Mets, including age and unhealthy lifestyles (such as reduced exercise and reduced intake of fruits and vegetables), are the possible reasons for the high risk of Mets among nurses in different departments. Meanwhile, our findings describe robust evidence for nurses to consider how psychological stress (more hours of work, work the night shift, bad self‐rated health status, and self‐assessment of inadequate sleep time) can increase the risk of Mets in hospital workers.^3^, ^4^, ^5^, ^6^, ^38^, ^39^, ^40^ Therefore, in addition to the traditional recommendation to prevent Mets (eg, healthy diet and physical activities), the individuals' psychosocial stress and occupational burnout syndrome are not been neglected, then the hospital workers can achieve the goal.

Another of the strengths of this study was we prospectively explored the biomarkers related to Mets and burnout. Our results focus on the predictive role of biomarkers, Hba1c, in Mets and burnout; but thyroid function from HPA axis had no significant relationship. The present data confirm that there are some psychological and physiological aspects related to stress in the nurse staff. Indeed, they may be relevant for further research in order to implement prevention programs aimed at reducing the negative aspects of professional distress. Importantly, for future research, using refined longitudinal studies is recommended, as these might be more useful and powerful in exploring causal relationships.

### 4.1 Limitations

A limitation of this study was that it was a single‐institution study. Another limitation of our study is that we cannot analyze the causality in the associations performed because the study design is cross‐section. Third limitation is that many factors (included severity and duration of burnout) are referred as added interpretations for evaluating the predicting function of the biomarkers that were needed to collect in further study.

## 5 CONCLUSIONS

We observed a significant association between burnout and Mets in nurses who work in a tertiary hospital. Other factors included seniority at work, night shifts, working hours, depressive mood, and poor self‐rated health and sleep status, which led to a higher risk of Mets. Unhealthy and sedentary lifestyle made the out‐patient nurse department had higher Mets risk compare with other nurses. Developing strategies to prevent burnout and Mets in nurses is necessary, especially those who have these risk factors. Agencies need to prioritize the improvement of traditional lifestyle habits and still need to incorporate stress management into current guidelines for preventing the Mets, especially in the hospital workplace.

## DISCLOSURES

*Approval of the research protocol:* The study protocol was examined and approved by the Human Research Ethics Committee at Mackay Memorial Hospital (project research number 18MMHISO150). *Informed consent:* Informed written consent was obtained from each participant before starting the study. *Registry and the registration no. of the study:* N/A. *Animal studies:* N/A. *Conflict of interest:* The authors declare no conflict of interests.

## AUTHOR CONTRIBUTIONS

MT Tsou participated in the design and conception of the study and its coordination, acquisition of data, carried out statistical analysis, and drafted the manuscript. TP Pai, TM Chiang participated in the conception of the study and participated in the design of the study, and reviewed analysis and manuscript. WH Huang, HM Lin, SC Lee participated in the design of the study, acquisition of data, and performed the statistical analysis.

## Supporting information

Supplementary Material
