# Supplementary material for: Burnout and metabolic syndrome among different departments of medical center nurses in Taiwan‐Cross‐sectional study and biomarker research
Source: J Occup Health. 2021 Jan 19;63(1):e12188. doi: 10.1002/1348-9585.12188 (PMC7815683; doi:10.1002/1348-9585.12188)
Supplement: Supplementary file 1 — Supplementary Material [file JOH2-63-e12188-s001.docx]

**Supplement table 1. Multivariate logistic regression analysis of burnout and working factors influencing Mets in different departments nurses**

| **Factors** |  | **Out-patient** | **General ward** | **Intensive care unit** | **Operation room** |
| --- | --- | --- | --- | --- | --- |
| Burnout | no (ref) | 1 | 1 | 1 | 1 |
|  | yes | 2.67 (1.03-6.95)* | 1.63 (0.61-4.38) | 4.46 (0.84-10.63) | 5.22 (0.13-17.51) |
| Age |  | 1.06 (1.01-1.12)* | 1.08 (1.02-1.13)* | 0.82 (0.64-1.05) | 1.28 (1.01-1.62)* |
| Education level | ≦Senior high school (ref) | 1 | 1 | 1 | 1 |
|  | College | 0.90 (0.29-2.77) | 0.91 (0.19-4.43) | 0.98 (0.68-1.40) | 0.96 (0.53-1.67) |
|  | ≧Graduate School | 0.41 (0.09-1.84) | 0.86 (0.16-4.61) | 0.92 (0.80-1.05) | 0.99 (0.72-1.18) |
| Seniority | < 2 years (ref) | 1 | 1 | 1 | 1 |
|  | 2-4 years | 0.83 (0.15-4.64) | 0.54 (0.22-1.34) | 0.60 (0.12-7.44) | 0.90 (0.24-5.98) |
|  | 4-10 years | 0.73 (0.17-3.21) | 0.70 (0.18-2.76) | 0.65 (0.19-2.19) | 0.92 (0.20-4.79) |
|  | >10yeras | 1.33 (0.30-5.87) | 1.39 (0.66-2.93) | 2.89 (0.29-9.86) | 1.12 (0.52-3.47) |
| Working hours/week | ≦ 45 hrs (ref) | 1 | 1 | 1 | 1 |
|  | 46-50 hrs | 1.01 (0.51-1.01) | 1.09 (0.53-2.27) | 1.22 (0.33-3.53) | 1.01 (0.23-2.54) |
|  | 51-59 hrs | 1.73 (0.31-9.68) | 1.31 (0.36-4.74) | 1.87 (0.56-6.99) | 1.24 (0.13-4.33) |
|  | ≧60 hrs | 1.85 (0.38-8.96) | 1.82 (0.80-4.12) | 2.32 (0.51-8.45) | 1.34 (0.49-5.38) |
| Work style | Regular class (ref) | 1 | 1 | 1 | 1 |
|  | Night shift | 4.12 (1.07-12.29)* | 3.60 (1.23-10.56)* | 1.73 (0.09-13.94) | 1.56 (0.23-8.35) |
|  | Three shifts | 3.57 (0.99-12.81) | 1.60 (0.73-3.55) | 0.84 (0.09-7.64) | 0.47 (0.09-2.37) |

*****Excluded the data of emergency and dialysis nurses (the Mets participant was less than 5 persons).

†Adjusted for sex, sleep time, smoking, alcohol, exercise, fruit, vegetable intake.

*p<0.05.

**Supplement table 2. The working factors, emotional status, and sleep condition in different departments**

|  |  |  | **Out-patient** | | |  | **Emergency** | | |  | **General ward** | | |  | **Intensive care unit** | | |  | **Operation room** | | |  | **Dialysis** | | |  | **Total** | | |  | **p value** |
| --- | --- | --- | --- | --- | --- | --- | --- | --- | --- | --- | --- | --- | --- | --- | --- | --- | --- | --- | --- | --- | --- | --- | --- | --- | --- | --- | --- | --- | --- | --- | --- |
| **Variables** | |  | **(n=375)** | | |  | **(n=129)** | | |  | **(n=865)** | | |  | **(n=241)** | | |  | **(n=113)** | | |  | **(n=35)** | | |  | **(n=1758)** | | |  |  |
| Seniority, n (%) | |  |  |  |  |  |  |  |  |  |  |  |  |  |  |  |  |  |  |  |  |  |  |  |  |  |  |  |  |  | <0.001 |
|  | < 2 years |  | 29 |  | 7.73% |  | 21 |  | 16.28% |  | 223 |  | 25.78% |  | 51 |  | 21.16% |  | 21 |  | 18.58% |  | 0 |  | 0.00% |  | 345 |  | 19.62% |  |  |
|  | 2-4 years |  | 24 |  | 6.40% |  | 30 |  | 23.26% |  | 147 |  | 16.99% |  | 60 |  | 24.90% |  | 13 |  | 11.50% |  | 2 |  | 5.71% |  | 276 |  | 15.70% |  |  |
|  | 4-10 years |  | 75 |  | 20.00% |  | 45 |  | 34.88% |  | 304 |  | 35.14% |  | 69 |  | 28.63% |  | 30 |  | 26.55% |  | 9 |  | 25.71% |  | 532 |  | 30.26% |  |  |
|  | >10yeras |  | 247 |  | 65.87% |  | 33 |  | 25.58% |  | 191 |  | 22.08% |  | 61 |  | 25.31% |  | 49 |  | 43.36% |  | 24 |  | 68.57% |  | 605 |  | 34.41% |  |  |
| Working hours/week, n (%) | |  |  |  |  |  |  |  |  |  |  |  |  |  |  |  |  |  |  |  |  |  |  |  |  |  |  |  |  |  | <0.001 |
|  | ≦ 45 hrs |  | 310 |  | 82.67% |  | 96 |  | 74.42% |  | 438 |  | 50.64% |  | 148 |  | 61.41% |  | 33 |  | 29.20% |  | 16 |  | 45.71% |  | 1041 |  | 59.22% |  |  |
|  | 46-50 hrs |  | 56 |  | 14.93% |  | 30 |  | 23.26% |  | 334 |  | 38.61% |  | 79 |  | 32.78% |  | 64 |  | 56.64% |  | 17 |  | 48.57% |  | 580 |  | 32.99% |  |  |
|  | 51-59 hrs |  | 7 |  | 1.87% |  | 1 |  | 0.78% |  | 65 |  | 7.51% |  | 11 |  | 4.56% |  | 15 |  | 13.27% |  | 2 |  | 5.71% |  | 101 |  | 5.75% |  |  |
|  | ≧60 hrs |  | 2 |  | 0.53% |  | 2 |  | 1.55% |  | 28 |  | 3.24% |  | 3 |  | 1.24% |  | 1 |  | 0.88% |  | 0 |  | 0.00% |  | 36 |  | 2.05% |  |  |
| Work style, n (%) | |  |  |  |  |  |  |  |  |  |  |  |  |  |  |  |  |  |  |  |  |  |  |  |  |  |  |  |  |  | <0.001 |
|  | Regular class |  | 345 |  | 92.00% |  | 36 |  | 27.91% |  | 163 |  | 18.84% |  | 47 |  | 19.50% |  | 39 |  | 34.51% |  | 13 |  | 37.14% |  | 643 |  | 36.58% |  |  |
|  | Night shift |  | 20 |  | 5.33% |  | 16 |  | 12.40% |  | 62 |  | 7.17% |  | 13 |  | 5.39% |  | 0 |  | 0.00% |  | 5 |  | 14.29% |  | 116 |  | 6.60% |  |  |
|  | Three shifts |  | 10 |  | 2.67% |  | 77 |  | 59.69% |  | 640 |  | 73.99% |  | 181 |  | 75.10% |  | 74 |  | 65.49% |  | 17 |  | 48.57% |  | 999 |  | 56.83% |  |  |
| BSRS-5 (mean ± SD) | |  | 4.75 | ± | 3.71 |  | 6.09 | ± | 4.22 |  | 6.19 | ± | 4.05 |  | 5.66 | ± | 3.65 |  | 4.96 | ± | 3.28 |  | 5.26 | ± | 3.48 |  | 5.71 | ± | 3.92 |  | <0.001 |
| BSRS-5, n (%) | |  |  |  |  |  |  |  |  |  |  |  |  |  |  |  |  |  |  |  |  |  |  |  |  |  |  |  |  |  | <0.001 |
|  | ≦5 |  | 254 |  | 67.73% |  | 72 |  | 55.81% |  | 457 |  | 52.83% |  | 137 |  | 56.85% |  | 77 |  | 68.14% |  | 24 |  | 68.57% |  | 1021 |  | 58.08% |  |  |
|  | 6-9 |  | 78 |  | 20.80% |  | 30 |  | 23.26% |  | 228 |  | 26.36% |  | 67 |  | 27.80% |  | 27 |  | 23.89% |  | 5 |  | 14.29% |  | 435 |  | 24.74% |  |  |
|  | 10-14 |  | 34 |  | 9.07% |  | 21 |  | 16.28% |  | 144 |  | 16.65% |  | 30 |  | 12.45% |  | 7 |  | 6.19% |  | 6 |  | 17.14% |  | 242 |  | 13.77% |  |  |
|  | ≧15 |  | 9 |  | 2.40% |  | 6 |  | 4.65% |  | 36 |  | 4.16% |  | 7 |  | 2.90% |  | 2 |  | 1.77% |  | 0 |  | 0.00% |  | 60 |  | 3.41% |  |  |
| Self-rated Health Status, n (%) | |  |  |  |  |  |  |  |  |  |  |  |  |  |  |  |  |  |  |  |  |  |  |  |  |  |  |  |  |  | 0.002 |
|  | Good |  | 116 |  | 30.93% |  | 36 |  | 27.91% |  | 186 |  | 21.50% |  | 71 |  | 29.46% |  | 29 |  | 25.66% |  | 5 |  | 14.29% |  | 443 |  | 25.20% |  |  |
|  | Common |  | 231 |  | 61.60% |  | 80 |  | 62.02% |  | 556 |  | 64.28% |  | 144 |  | 59.75% |  | 73 |  | 64.60% |  | 28 |  | 80.00% |  | 1112 |  | 63.25% |  |  |
|  | Bad |  | 28 |  | 7.47% |  | 13 |  | 10.08% |  | 123 |  | 14.22% |  | 26 |  | 10.79% |  | 11 |  | 9.73% |  | 2 |  | 5.71% |  | 203 |  | 11.55% |  |  |
| Sleep duration, hours (mean, SD) | |  | 6.551 | ± | 0.87 |  | 6.87 | ± | 1.26 |  | 6.63 | ± | 1.18 |  | 6.58 | ± | 1.06 |  | 6.42 | ± | 0.99 |  | 6.62 | ± | 1.12 |  | 6.61 | ± | 1.10 |  | 0.04 |
| Self-assessment of sleep time | |  |  |  |  |  |  |  |  |  |  |  |  |  |  |  |  |  |  |  |  |  |  |  |  |  |  |  |  |  | <0.001 |
|  | Adequate |  | 184 |  | 49.07% |  | 55 |  | 42.64% |  | 271 |  | 31.33% |  | 93 |  | 38.75% |  | 40 |  | 35.71% |  | 12 |  | 34.29% |  | 655 |  | 37.30% |  |  |
|  | Inadequate |  | 191 |  | 50.93% |  | 74 |  | 57.36% |  | 594 |  | 68.67% |  | 147 |  | 61.25% |  | 72 |  | 64.29% |  | 23 |  | 65.71% |  | 1101 |  | 62.70% |  |  |
| Chinese occupational burnout inventory (mean, SD) | |  |  |  |  |  |  |  |  |  |  |  |  |  |  |  |  |  |  |  |  |  |  |  |  |  |  |  |  |  |  |
|  | Psychological work demands |  | 67.70 | ± | 17.40 |  | 79.74 | ± | 14.71 |  | 76.45 | ± | 16.09 |  | 73.44 | ± | 15.68 |  | 81.06 | ± | 15.29 |  | 67.43 | ± | 15.99 |  | 67.43 | ± | 15.99 |  | <0.001 |
|  | Job control |  | 54.94 | ± | 11.51 |  | 54.03 | ± | 11.51 |  | 58.03 | ± | 10.37 |  | 60.14 | ± | 10.10 |  | 54.74 | ± | 11.87 |  | 53.33 | ± | 9.72 |  | 57.06 | ± | 10.98 |  | <0.001 |
|  | Employment stability |  | 54.49 | ± | 15.02 |  | 59.43 | ± | 15.18 |  | 63.26 | ± | 14.43 |  | 64.32 | ± | 13.97 |  | 59.07 | ± | 13.75 |  | 57.14 | ± | 11.46 |  | 60.86 | ± | 14.91 |  | <0.001 |
| Maslach Burnout Inventory (MBI) (Mean, SD) | |  |  |  |  |  |  |  |  |  |  |  |  |  |  |  |  |  |  |  |  |  |  |  |  |  |  |  |  |  |  |
|  | Emotional exhaustion |  | 23.19 | ± | 10.54 |  | 28.24 | ± | 11.11 |  | 29.32 | ± | 10.50 |  | 26.79 | ± | 9.95 |  | 26.40 | ± | 10.40 |  | 25.15 | ± | 10.39 |  | 27.31 | ± | 10.74 |  | <0.001 |
|  | Depersonalization |  | 9.55 | ± | 5.39 |  | 12.18 | ± | 5.99 |  | 12.46 | ± | 5.98 |  | 11.30 | ± | 5.67 |  | 11.32 | ± | 5.00 |  | 8.79 | ± | 4.89 |  | 11.51 | ± | 5.86 |  | <0.001 |
|  | Personal accomplishment |  | 26.37 | ± | 5.52 |  | 25.94 | ± | 5.54 |  | 27.98 | ± | 4.98 |  | 28.87 | ± | 4.85 |  | 26.28 | ± | 5.70 |  | 25.60 | ± | 4.67 |  | 27.39 | ± | 5.27 |  | <0.001 |

* n= numbers, SD= Standard deviation, BSRS= Brief Symptom Rating Scale
